# Integrated hearing and genetic screening for neonatal deafness in a resource-limited region: insights from Qingyuan, China

**DOI:** 10.3389/fgene.2026.1873535

**Published:** 2026-06-30

**Authors:** Qin She, Hui-Lin Ou, Er-Fang Tang, Wei Huang, Wei-He Tan, Mu-Lan Zeng, Xiu-Feng Pan, Chen-Bing Liu

**Affiliations:** 1 Prenatal Diagnostic Center, The Affiliated Qingyuan Hospital (Qingyuan People’s Hospital), Guangzhou Medical University, Qingyuan, Guangdong, China; 2 Department of Obstetrics and Gynecology, Kaifeng Maternal and Child Health Hospital, Kaifeng, Henan, China

**Keywords:** genetic screening for deafness, GJB2 gene mutations, neonatal hearing screening, resource-limited regions, whole exome sequencing (WES)

## Abstract

**Objective:**

This study aimed to delineate the prevalence and mutation spectrum of deafness-associated genes among newborns in a resource-limited region of China, and to assess the diagnostic yield of combined hearing and genetic screening.

**Methods:**

From May 2017 to May 2023, 22,819 newborns underwent concurrent hearing screening and genetic screening for deafness-associated mutations using 9-variant or 23-variant microarrays targeting GJB2, SLC26A4, GJB3, and mitochondrial 12SrRNA. Whole-exome sequencing was performed in selected cases with inconclusive results. All infants were followed until 2.5 years of age.

**Results:**

Hearing screening confirmed hearing loss in 25 infants (0.11%). Genetic screening identified 994 (4.36%) mutation carriers, predominantly GJB2 (3.31%) and SLC26A4 (0.78%). Among 351 GJB2 109G>A heterozygotes, all had normal hearing at 2.5 years. Among 43 homozygotes, 7 (16.28%) had congenital deafness; the rest remained normal. All 42 mitochondrial mutation carriers remained deafness-free after preventive education. The 23-variant panel detected 24.73% positive cases (225-fold vs. hearing alone). WES resolved 80% of ambiguous cases.

**Conclusion:**

GJB2 and SLC26A4 are the predominant deafness genes in this region. Combined screening identifies distinct risk groups, heterozygotes for reproductive counselling, mitochondrial carriers for prevention, and homozygotes for early intervention. Long-term follow-up of normal-hearing homozygotes showed low short-term delayed-onset risk.

## 1 Introduction

Hearing loss, a complex sensory disorder affecting over 1.5 billion people worldwide, arises from structural or functional abnormalities in the auditory system (Pan et al., 2024; Shearer and Smith, 2015; Mahboubi et al., 2012). Genetic mutations and chromosomal anomalies account for >50% of congenital cases, with non-syndromic hearing loss (NSHL, 70%) predominating over syndromic forms (SHL, 30%) (Shearer and Smith, 2015; Elsayed and Al-Shamsi, 2022; Shi et al., 2024; Marazita et al., 1993; Sheffield and Smith, 2019). Environmental factors such as prenatal infections, ototoxic medications, and noise exposure further compound this burden, particularly in low-resource settings (Sheffield and Smith, 2019). The World Health Organization estimates that 430 million individuals currently require rehabilitation for disabling hearing loss, underscoring its status as a critical public health challenge.

Previously, hearing screening has been the primary method for detecting neonatal hearing impairment. However, as screening programs have been widely implemented and clinical experience has accumulated, it has gradually become evident that hearing screening alone cannot identify newborns with late-onset genetic hearing loss or those carrying defective deafness-associated genes (Wroblewska-Seniuk et al., 2017). With the rapid advancement in cloning techniques for hereditary deafness-related genes and the development of molecular epidemiological research, genetics has been recognized as the leading cause of hearing loss, accounting for over 60% of cases. In China, since 2003, the Molecular Diagnostic Center for Deafness at the PLA General Hospital took the lead in conducting nationwide molecular epidemiological surveys on hearing loss. The results indicated that genes such as GJB2, SLC26A4, mitochondrial genes, and GJB3 are the most common deafness-associated genes in China (Dai et al., 2019).

Although these nationwide epidemiological studies have revealed the mutation spectrum of deafness genes in the Chinese population and laid a scientific foundation for subsequent screening efforts, translating this scientific knowledge into equitable public health practice remains a significant challenge. Over the past 2 decades, screening practices have largely remained a “geographical privilege,ˮ highly concentrated in cities with abundant medical resources such as Beijing, Shanghai, Guangzhou, and Ningbo. In stark contrast, in vast economically underdeveloped regions, particularly remote mountainous areas with poor transportation, the coverage of neonatal hearing screening is still insufficient, and genetic screening for deafness, which involves higher costs and technical barriers, remains nearly non-existent. This severe imbalance in screening resources has led to a large number of newborns carrying pathogenic genes in economically disadvantaged areas being excluded from early intervention systems.

Qingyuan, located in the mountainous area in northern Guangdong Province, is one of the least developed regions in China. Over 50% of its population resides in remote rural areas with limited healthcare resources and lower socioeconomic status. These factors pose significant challenges to the implementation of neonatal screening programs. Despite the high prevalence of genetic deafness in China, data from economically disadvantaged regions like Qingyuan remains scarce, which poses great difficulties for the tertiary prevention of deafness in the local area.

As the only tertiary comprehensive hospital in Qingyuan area, Qingyuan People’s Hospital is the critical care center for pregnant and postpartum women in Qingyuan area. The annual delivery newborns exceeds 7,000, accounting for about 20% of the total deliveries in Qingyuan area. This study aimed to delineate the prevalence and mutation spectrum of deafness-associated genes among newborns in Qingyuan, a resource-limited region of China, and to assess the diagnostic yield of combining genetic screening with conventional newborn hearing screening. By characterizing the local genetic epidemiology, we seek to provide a foundational evidence base for future public health initiatives.

## 2 Materials and methods

### 2.1 Study population and ethical considerations

A total of 22,819 newborns delivered at Qingyuan People’s Hospital between May 2017 and May 2023 were enrolled in this cohort study. The study protocol was approved by the Institutional Ethics Committee of Qingyuan People’s Hospital (Approval No. IRB-2025-160). Written informed consent was obtained from all guardians prior to participation.

### 2.2 Hearing screening

#### 2.2.1 Primary hearing screening

Newborns undergo hearing screening from 48 h after birth to before discharge. Transiently evoked otoacoustic emission (TEOAE; Interacoustics Titan, Denmark) were performed in quiet environments (<40 dB ambient noise) in General Wards. Combined automated auditory brainstem response (AABR; Natus ALGO 3i, United States) and TEOAE were used for high-risk infants in Neonatal Intensive Care Units (NICU).

#### 2.2.2 Rescreening

Infants failing the initial screening underwent AABR retesting at 42 days postpartum, adhering to the Joint Committee on Infant Hearing (JCIH) guidelines.

### 2.3 Genetic screening for deafness

#### 2.3.1 Sample collection and processing

Dried blood spots were collected from all Infants. Umbilical cord blood (2 mL) and peripheral blood were collected for families that require further whole exome sequencing. DNA extraction using the QIAamp DNA Blood Mini Kit (Qiagen, Germany).

#### 2.3.2 Targeted gene panel testing

The Deafness Gene Variant Detection Array Kit (CapitalBio) was used to identify nine/twenty three variants in four genes, including GJB2, SLC26A4, GJB3, and mitochondrial 12SrRNA. Genetic mutations were screened using two DNA microarray platform (Table 1).

**TABLE 1:** Common deafness gene mutation sites detected by different screening kits.

Gene	Mutation sites (9-variant kit)	Mutation sites (23-variant kit)
GJB2	235delC, 299delAT, 176del16,35delG	235delC,299delAT,176del16,35delG,109G>A,257C>G,512insAACG,427C>T,35insG
SLC26A4	IVS7-2A>G,2168A>G	2168A>G,917insG,1707 + 5G>A,589G>A,2027T>A,1229C>T,1226G>A,1174A>T,919-2A>G,1975G>C,281C>T
12SrRNA	1555A>G,1494C>T	1555A>G, 1494C>T
GJB3	538C>T	538C>T

The 23 variant Kit contains all 9-variant Kit loci.

The 919-2A>G locus in the 23 variant Kit is the same as the IVS7-2A>G locus in the 9-variant Kit loci.

Phase 1 (May 2017–August 2022): the nine variants (9-variant) was used to screen 9 loci across four genes: GJB2, SLC26A4, GJB3, and mitochondrial 12SrRNA.

Phase 2 (September 2022–May 2023): the twenty three variants (23-variant) was used to screen 23 loci across four genes: GJB2, SLC26A4, GJB3, and mitochondrial 12SrRNA.

### 2.4 Whole exome sequencing

This study whole exome sequencing (WES) was employed for infants meeting any of the following to analyze fetal-parental trio samples for genetic investigations: persistent hearing screening failure after rescreening; negative or heterozygous results on initial genetic screening.

The experimental workflow strictly followed standardized protocols: Exonic sequences were enriched in the DNA sample using Agilent SureSelect human exome capture probes (V6, Life Technologies). The DNA library was sequenced on Illumina Novaseq 6000 system (Illumina, Inc.) to obtain 150 bp paired-end reads. Coverage for the samples was >99% at a 20x depth threshold.

Raw data underwent quality control using FastQC, followed by alignment to the GRCh37 reference genome. Detected variants were functionally annotated using Annovar and InterVar, and classified according to the American College of Medical Genetics and Genomics (ACMG) guidelines into five categories: pathogenic (P), likely pathogenic (LP), variant of uncertain significance (VUS), likely benign, and benign. For fetal samples, co-segregation analysis was performed under multiple inheritance models (autosomal recessive/dominant, X-linked, and *de novo* mutations), and critical variants were validated by Sanger sequencing.

### 2.5 Follow-up

All cases included in this study were followed up. All newborns were referred to the pediatric healthcare department at 42 days after birth for regular follow-up observations until 2.5 years of age. Infants who did not pass the primary hearing screening underwent rescreening at 42 days after birth. Those who still did not pass the rescreening were referred to the otolaryngology department for further management. Newborns with abnormal deafness gene screening results received further genetic counseling and health education (see Supplementary Table S1). For infants carrying mitochondrial 12SrRNA mutations, specific health education regarding aminoglycoside avoidance was provided to families. For infants with biallelic GJB2 mutations who passed neonatal hearing screening, regular audiological monitoring was conducted at 6, 12 and 24 months of age.

### 2.6 Statistical methods

Statistical analysis was conducted using SPSS 25.0. The statistical description of quantitative data is expressed as mean ± standard deviation, while qualitative data is expressed as frequency and percentage. The statistical analysis of quantitative data uses t-test based on whether the sample is normally distributed, while qualitative data uses chi square test. *P* < 0.05 (two-tailed) indicates that the difference is statistically significant.

## 3 Result

### 3.1 Hearing screening

**FIGURE 1:**
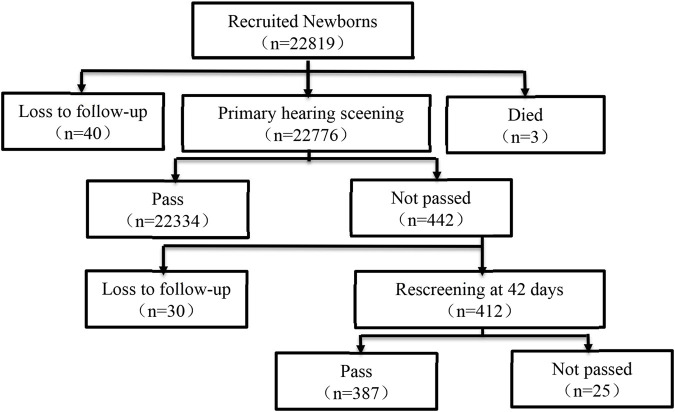
Flowchart of newborn hearing screening results.

A total of 22,819 newborns were enrolled in this study, with 22,776 (99.81%) completing primary hearing screening. The initial screening failure rate was 1.94% (442/22,776). Among these 442 infants, 412 underwent rescreening at 42 days postpartum, yielding a 93.93% pass rate (387/412) and a 6.07% persistent failure rate for hearing screening either bilaterally or unilaterally (25/412) (Table 2). After accounting for 30 cases lost to follow-up, the final prevalence of confirmed hearing loss was 0.11% (25/22,746). Notably, logistical challenges, such as poverty, parental cognitive issues, or limited transportation infrastructure in remote villages, contributed to delayed or missed screenings in 0.32% of cases (73/22,819) (Figure 1).

**TABLE 2:** Results of hearing screening.

Results	Number of cases	Percentage (%)
Primary hearing screening (n = 22,776)		
Pass	22,334	98.06
Unilateral refer	273	1.20
Bilateral refer	169	0.74
Second hearing screening (n = 412)		
Pass	387	93.93
Unilateral refer	6	1.46
Bilateral refer	19	4.61

### 3.2 Genetic screening for neonatal hearing loss

#### 3.2.1 Overall mutation profile

Genetic screening identified 994 newborns (4.36%, 994/22,819) with deafness-associated mutations. The GJB2 gene exhibited the highest mutation frequency (3.31%, 756/22,819), followed by SLC26A4 (0.78%, 179/22,819). Statistical analysis revealed significant differences in mutation prevalence across genes (χ^2^ = 2069.94, *P* < 0.001) (Table 3). Among the 994 newborns with positive genetic screening results, 903 were carriers with normal hearing, 42 carried mitochondrial 12SrRNA mutations and remained hearing-normal after preventive education, 1 had a homozygous GJB2 235delC mutation with congenital hearing loss, 43 had a homozygous GJB2 109G>A mutation (7 with hearing impairment at birth), and 5 had compound heterozygous GJB2 109G>A mutations (1 with unilateral deafness at birth).

**TABLE 3:** Detection status of deafness gene in newborns (n = 22,819).

Gene	Positive(n)	Percentage (%)
GJB2	756	3.31
SLC26A4	179	0.78
GJB3	13	0.06
12S rRNA	42	0.18
GJB2 and SLC26A4	4	0.02
Total	994	4.36
χ2	​	2069.94
*P*	​	0.00

#### 3.2.2 Performance of genetic screening kits

**FIGURE 2:**
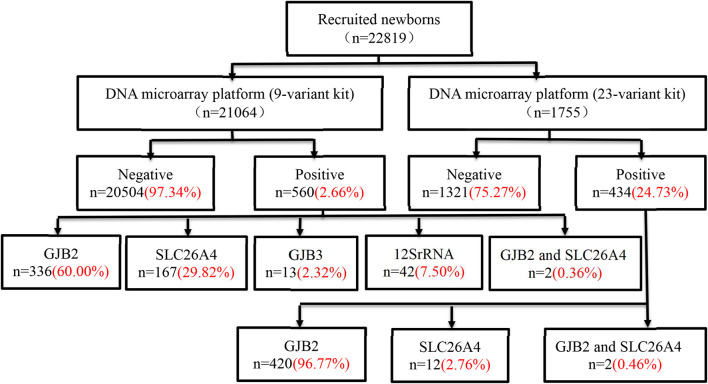
Detection of deafness genes using two kits.

9-Variant Kit (May 2017–August 2022): Screened 21,064 newborns, detecting 560 positive cases (2.66%), predominantly GJB2 and SLC26A4 (Figure 2).

23-Variant Kit (September 2022–May 2023): Screened 1,755 newborns, identifying 434 positive cases (24.73%), with higher yields for GJB2and SLC26A4 (Figure 2).

The expanded 23-variant panel significantly outperformed the 9-variant kit (χ^2^ = 1894.13, *P* < 0.001) (Table 4).

**TABLE 4:** Detection status of two types of deafness gene detection kits.

Variants	9-Variant kit			23-Variant kit			Total(n)
	Number of cases	Percentage (%) (n = 560)	Percentage (%) (n =21,064)	Number of cases	Percentage (%) (n = 434)	Percentage (%) (n = 1,755)	
Refer							
GJB2 homozygote	**1**	**0.18**	**<0.01**	**43**	**9.91**	**2.45**	**44**
GJB2 235delC	1	0.18	**<0.01**	0	0	0	1
GJB2 109G>A	—	—	​	43	9.91	2.45	43
GJB2 compound heterozygote	**0**	**0**	**0**	**5**	**1.15**	**0.28**	**5**
GJB2 109G>A with GJB2 235delC	—	—	—	4	0.92	0.23	4
GJB2 109G>A with GJB2 299delAT	—	—	—	1	0.23	0.06	1
12S rRNA m.1555A>G	**36**	**6.43**	**0.17**	**0**	**0**	**0**	**36**
12S rRNA m.1494 C>T	**6**	**1.07**	**0.03**	**0**	**0**	**0**	**6**
Total	**43**	**7.68**	**0.21**	**48**	**11.06**	**2.74**	**91**
Carrier							
GJB2 heterozygote	335	59.82	1.59	373	85.94	21.25	708
SLC26A4 heterozygote	167	29.82	0.79	12	2.76	0.68	179
GJB3 heterozygote	13	2.32	0.06	0	0	0	13
GJB2 heterozygote with SLC26A4 heterozygote	2	0.36	<0.01	1	0.23	0.06	3
Total	**517**	**92.32**	**2.45**	**386**	**88.94**	**21.99**	**903**
Total for all screenings							
Total	**560**	​	​	**434**	​	​	—

"‐" means no cases were identified. Bold values represent the total number of cases and percentages within the “Refer” and “Carrier” subgroups, as well as the overall totals for the 9‐variant and 23‐variant kits.

#### 3.2.3 GJB2 gene

In this study, GJB2 gene screening was performed on all 22,819 neonates, and 760 were found to carry variants of this gene. Among them, 338 were detected by the 9-Variant Kit, with the GJB2 235delC variant being the most prevalent, accounting for approximately 79.88% (270/338). The remaining 422 were detected by the 23-Variant Kit, where the GJB2 109 G>A variant was the most common, representing about 94.55% (399/422), followed by the GJB2 235delC variant (4.27%, 18/422) Supplementary Table S2.

Among the 399 neonates carrying the GJB2 109G>A variant, 351 had a heterozygous mutation, and all passed the hearing screening. At 2.5-year follow-up, all 351 heterozygous carriers remained hearing-normal, confirming that heterozygous carrier status does not confer increased risk of hearing loss. Forty-three had a homozygous mutation: 7 (16.28%, 7/43) exhibited hearing impairment at birth, the remaining 36 remained hearing-normal at 2.5-year follow-up. Five had compound heterozygous mutations: 1 (20.00%, 1/5) presented with unilateral deafness at birth, and the remaining 4 remained hearing-normal at 2.5-year follow-up.

#### 3.2.4 Mitochondrial gene

In this study, all 22,819 neonates were tested for the 12SrRNA gene. A total of 42 cases (0.18%, 42/22,819) were identified with 12SrRNA variants. All of these infants passed the initial hearing screening. All 42 families received comprehensive health education regarding aminoglycoside ototoxicity and the importance of avoiding these medications. At 2.5-year follow-up, none of the 42 children had developed drug-induced deafness, demonstrating the effectiveness of preventive education in this high-risk population.

#### 3.2.5 Combined screening efficacy

In this study, excluding 73 newborns who were unable to complete primary hearing screenings and rescreening at 42 days postpartum due to loss of follow-up or death, a total of 22,746 newborns underwent combined hearing and deafness gene screening. Among them, 20,991 received the 9-Variant Kit combined hearing screenings, while 1,755 received the 23-Variant Kit combined hearing screenings.

Integrated hearing and genetic screening of 22,746 newborns achieved an overall detection rate of 4.43% (1,008/22,746). Stratified analysis demonstrated 2.74% detection rate (575/20,991) in 9-Variant Kit combined hearing screening group, while 24.73% detection rate (434/1,755) in 23-Variant Kit combined Hearing Screening group, reflecting a 9-fold increase in diagnostic yield (*P* < 0.001) (Table 5).

**TABLE 5:** Detection status of two types of deafness gene detection kits combined with hearing screening.

Screening protocol	Positive (n/%)	Negative (n/%)	Total (n)
9-Variant kit combined hearing screening	575 (2.74)	20,416 (97.26)	20,991
23-Variant kit combined hearing screening	434 (24.73)	1,321 (75.27)	1755
Total (n)	1,009	21,737	22,746
χ2	​	​	1847.47
*P*	​	​	<0.001

### 3.3 Whole exome sequencing (WES) findings

In this study, a total of 25 infants failed both the primary hearing screening and rescreening at 42 days after birth. Among these, 9 cases were identified with causative factors through deafness gene screening. Specifically, 7 cases involved a homozygous GJB2 109G>A mutation, 1 case involved a homozygous GJB2 235 del C mutation, and 1 case involved a compound heterozygous mutation (case 21:GJB2 235delC with GJB2 109G>A). The remaining 16 cases had no identifiable etiology Supplementary Table S3.

Among 16 neonates with abnormal hearing screening results but inconclusive genetic findings (negative or heterozygous mutations), five underwent trio-based WES with parental consent. Pathogenic or likely pathogenic variants were identified in 80% (4/5) of these cases (Table 6), demonstrating the critical role of WES in resolving diagnostically challenging scenarios.

**TABLE 6:** Pathogenic mutations identified by whole exome sequencing.

Case	Gene	Chromosome	Transcript	Nucleotide change	Amino acid change	Zygosity	Inheritance	Pathogenicity	Origin
1	STRC	15	NM_153700	Exon 16–19 duplication	—	Heterozygous	AR	Uncertain	Paternal
				c.2303_2313+1del12	—	Heterozygous	AR	Likely pathogenic	Maternal
2	GJB2	13	NM_004004	c.109G>A	p.Val37Ile	Homozygous	AD/AR	Pathogenic	Both
3	GJB2	13	NM_004004	c.235delC	p.Leu79CysfsTer3/p.Ala171GlufsTer40	Heterozygous	AD/AR	Pathogenic	Maternal
			NM_004004	c.508_511dupAACG	p.Ala171GLUfsTer40	Heterozygous	AD/AR	Pathogenic	Paternal
4	KAT6B	10	NM_012330.3	c.5385C>A	p.Tyr1795Ter	Heterozygous	AD	Pathogenic	De novo

Case 1 A newborn with bilateral hearing screening failure and negative panel results was diagnosed with congenital hearing loss at 12 months. WES revealed a heterozygous STRC deletion (c.2303_2313+1del12) and exon 16–29 repeats, prompting bilateral cochlear implantation.

Case 2 Despite negative panel screening, WES identified a homozygous GJB2 variant (c.109G>A, p.Val37Ile), a well-characterized pathogenic mutation in autosomal recessive deafness. The infant received bilateral cochlear implants.

Case 3 Initial testing showed a heterozygous GJB2 c.235delC mutation. WES uncovered a compound heterozygous genotype (c.235delC + c.508_511dupAACG), confirming autosomal recessive deafness. The child received a right cochlear implant and left hearing aid.

Case 4 WES detected a *de novo* heterozygous KAT6B nonsense variant (c.5385C>A, p.Tyr1795Ter), associated with syndromic hearing loss. The infant died to complications at 7 months.

Case 5 No pathogenic variants were detected by WES, yet bilateral cochlear implantation was pursued empirically due to persistent auditory neuropathy.

## 4 Discussion

### 4.1 Public health burden and genetic basis of deafness

Hearing loss, a major contributor to global disability, profoundly impacts speech development, educational attainment, and social integration (GBDHL, 2021; Zhu et al., 2022). Over 50% of congenital deafness cases are attributable to genetic factors, with non-syndromic hearing loss (70%) predominating over syndromic forms (30%) (Hoefsloot et al., 2014). While newborn hearing screening remains the cornerstone for early detection, its standalone efficacy is limited by false negatives and inability to identify late-onset genetic hearing loss. Integrating molecular screening for deafness-associated genes addresses these gaps, enabling proactive interventions and risk stratification.

### 4.2 Regional disparities in screening outcomes

This study identified a hearing screening positivity rate of 0.11% in Qingyuan, markedly lower than rates reported in Beijing (1.06%) (Dai et al., 2019). This discrepancy likely reflects socioeconomic and geographic barriers unique to resource-limited regions (Chen et al., 2012; Wen and Huang, 2023). Rural populations in Qingyuan face challenges such as limited healthcare access, follow-up losses due to distance from hospitals, and insufficient awareness of preventive care, factors that compromise screening adherence. These findings highlight the need for tailored screening strategies that account for regional infrastructure and population characteristics.

### 4.3 Genetic epidemiology and mutation profiles

Notably, the overall deafness gene mutation rate in Qingyuan (4.36%) is basically consistent with that of Ningbo (4.32%), Beijing (4.9%), and other regions (Wen et al., 2023), underscoring genetic homogeneity among Han Chinese populations. GJB2 (3.31%) and SLC26A4 (0.78%) emerged as the most prevalent mutated genes, consistent with nationwide cohorts (Liu et al., 2023; Feng et al., 2024; Zhang et al., 2023). These findings reinforce their prioritization in population-level screening programs.

Deafness caused by GJB2 gene mutations is the most common form of hereditary deafness, and effective therapeutic drugs have long been lacking. In this study, we performed GJB2 gene screening on all 22,819 neonates, identifying 760 carriers of the gene, resulting in a carrier rate of approximately 3.33% (760/22,819). This rate was higher than that reported by Feng J, et al. (Feng et al., 2024), which may be attributed to the inclusion of more GJB2 loci in our study.

The GJB2 109G>A mutation, due to its high variability in clinical phenotypes and incomplete penetrance, has been debated regarding its inclusion in routine screening. However, our longitudinal data demonstrate that screening for this locus in newborns holds significant importance for risk stratification and clinical management. The 23-Variant Kit screened 1,755 neonates for the GJB2 109G>A mutation, revealing a mutation rate of 22.74%, consistent with literature reports (Zou et al., 2019). The proportion of hearing impairment present at birth among those with the GJB2 109G>A homozygous mutation was 16.28%, aligning with literature reports (Shen et al., 2019). Similarly, the proportion of hearing impairment present at birth among those with compound heterozygous GJB2 109G>A mutations was 20.00%, also consistent with published data (Zou et al., 2019). Literature studies have confirmed that homozygous and compound heterozygous GJB2 109G>A mutations not only cause congenital hearing loss in some neonates but are also closely associated with delayed-onset hearing loss. A Shenzhen study of 652 newborns with biallelic GJB2 mutations showed that 8 infants who passed newborn hearing screening were diagnosed with hearing loss at 2.5–6 months of age, confirming the risk of delayed onset (Li et al., 2025). The ClinGen Expert Panel consensus indicates that this variant exhibits age-related penetrance, with an estimated hearing loss progression of approximately 1 dB per year (Shen et al., 2019). Mechanistic studies suggest that the p. V37I variant impairs gap junction protein function, rendering the cochlea susceptible to damage and leading to progressive hearing loss during development (Chen and Jiang, 2024). These literature findings further support the necessity of including the GJB2 109G>A variant in newborn genetic screening, which enables long-term audiological surveillance for infants with normal neonatal hearing but at risk for delayed onset, thereby preventing delayed diagnosis and missed opportunities for intervention during the critical window for language development.

For GJB2 109G>A heterozygous carriers (n = 351), all remained hearing-normal at 2.5-year follow-up. This confirms that heterozygous carrier status may not confer in-creased risk of hearing loss and provides reassurance to families. However, the identification of carrier status offers important reproductive counseling value: families learn of their risk for having affected children in future pregnancies, enabling informed reproductive choices including prenatal diagnosis or preimplantation genetic testing.

### 4.4 Mitochondrial gene

Mitochondrial DNA mutations have been identified as being associated with both syndromic and non-syndromic hearing loss inherited maternally worldwide. The 12SrRNA gene is a mitochondrial deafness-related gene. Children carrying mutations in this gene may experience hearing loss when exposed to aminoglycosides, such as gentamicin, kanamycin, and streptomycin, due to the binding of the mitochondrial gene to the drug, a condition commonly referred to as the “one-shot deafness gene.' The ototoxicity of aminoglycosides remains inadequately recognized in developing countries, leading to frequent cases of permanent, bilateral sensorineural hearing loss in children treated with aminoglycosides for other conditions, such as tuberculosis or bacterial infections (Ding et al., 2013).

In this study, 0.18% of newborns were found to carry 12S rRNA mutations. All passed the hearing screening at birth, and following health education based on the screening results, none developed drug-induced deafness by 2.5 years of age. This demonstrates that genetic screening, when coupled with appropriate counseling and education, can prevent iatrogenic disease, a particularly important outcome in regions where aminoglycoside use may be less regulated.

### 4.5 Synergy of combined screening strategies

In this study, the detection rate based on hearing screening alone was only 0.11%. However, when hearing screening was combined with the 23-variant deafness gene screening kit, the detection rate increased by 225-fold. This substantial increase reflects the fact that genetic screening identifies not only infants with congenital hearing loss but also asymptomatic carriers (reproductive counseling value) and preventable-risk infants (mitochondrial mutation carriers).

The substantial improvement in screening efficiency primarily stems from the complementary nature of the two approaches: hearing screening can only identify existing hearing loss at birth, whereas genetic screening provides early warning of potential delayed-onset hearing loss and identifies high-risk individuals susceptible to ototoxic drugs, and provides reproductive risk information for families. Thus, the combined strategy achieves comprehensive coverage of current status, future risk, and family-level genetic information.

### 4.6 Role of whole exome sequencing (WES)

In cases with ambiguous results (heterozygous mutations or negative result in genetic screening kits), WES identified pathogenic variants in 80% (4/5) of infants, including STRC mutations and KAT6B nonsense mutations. This aligns with global trends advocating WES as a second-tier test for unresolved cases, particularly in genetically heterogeneous conditions like deafness. By uncovering rare or novel variants, WES enhances diagnostic precision, enabling tailored management plans. In resource-limited regions, while WES may not be feasible as a first-line test, its targeted use in cases with strong clinical suspicion but negative panel results can provide definitive diagnoses and guide management, as demonstrated in this study.

### 4.7 Limitations and future directions

Despite its contributions, this study has several limitations. First, recruitment from a single tertiary hospital may not fully represent rural populations in the region, introducing potential single-center bias. Second, although follow-up to 2.5 years of age pro-vides valuable longitudinal data, some forms of late-onset hearing loss, particularly those associated with certain genotypes,may manifest after this age; extended follow-up through school age is therefore needed. Third, while whole-exome sequencing and expanded gene panels improve diagnostic detection, formal cost-effectiveness analyses in resource-limited settings are required to guide policy decisions. Fourth, the benefits of carrier detection for reproductive counseling and preventive education for mitochondrial mutation carriers depend on adequate genetic counseling infrastructure, which may be limited in resource-constrained regions.

### 4.8 Implications for public health

This study provides a scalable blueprint for deafness prevention in resource-limited regions. By coupling portable genetic technologies with primary care networks, regions like Qingyuan can mitigate infrastructure constraints while achieving early diagnosis rates comparable to affluent areas. These findings advocate for policy reforms to integrate combined screening into national newborn health programs, ultimately reducing the lifelong burden of hearing loss.

## Data Availability

The original contributions presented in the study are included in the article/Supplementary Material, further inquiries can be directed to the corresponding author.
