# Supplementary material for: Integrated hearing and genetic screening for neonatal deafness in a resource-limited region: insights from Qingyuan, China
Source: Front Genet. 2026 Jun 30;17:1873535. doi: 10.3389/fgene.2026.1873535 (PMC13365855; doi:10.3389/fgene.2026.1873535)
Supplement: Supplementary file 1 [file Table1.docx]

**Table S1 Health Education**

| **Gene** | **Type of Onset** | **Lifestyle Guidance** |
| --- | --- | --- |
| **GJB2** | Moderate, severe, profound deafness；  Conductive hearing loss；  Sensory neural hearing loss | 1.Avoid consanguineous marriage; undergo genetic testing for deafness in spouses before marriage and pregnancy; conduct prenatal genetic testing for deafness in the fetus.  2.Deafness caused by genetic mutations is typically congenital, but in some cases, it may not present at birth and can manifest as delayed-onset hearing loss.  3.Conduct regular hearing tests, follow medical advice, seek prompt medical attention when hearing loss occurs, and promptly use hearing aids or undergo cochlear implantation to prevent the decline of language function. |
| **SLC26A4** | Moderate, severe, profound deafness；  Sensory neural hearing loss | 1.Avoid consanguineous marriage; undergo genetic testing for deafness in spouses before marriage and pregnancy; conduct prenatal genetic testing for deafness in the fetus.  2.Undergo temporal bone CT for confirmation and receive timely medical treatment.  3.Strictly avoid head trauma, refrain from participating in intense sports, avoid head-down positions, try to prevent colds, and do not blow the nose or cough forcefully.  4.Avoid ototoxic drugs and stay away from noise.If hearing loss recurs or worsens, seek medical attention promptly. |
| **12SrRNA** | Moderate, severe, profound deafness；  Sensory neural hearing loss | Aminoglycoside antibiotics, including the following, should be avoided: streptomycin, kanamycin, tobramycin, spectinomycin, neomycin, gentamicin, vindemycin, sisomicin, micronomicin, astromicin, amikacin, netilmicin, ribostamycin, etimicin sulfate, isepamicin sulfate, and gentamicin sulfate. |
| **GJB3** | Acquired high-frequency sensorineural hearing loss | 1.Avoid consanguineous marriage; undergo genetic testing for deafness in spouses before marriage and pregnancy; conduct prenatal genetic testing for deafness in the fetus.  2.Pay attention to acquired high-frequency hearing loss, undergo regular hearing tests, follow medical advice, and seek prompt medical attention at a hospital if hearing loss occurs. |

**Table S2 GJB2 pathogenic variants**

| **GJB2 Gene** | **Total** | **9-variant Kit** | **23-variant Kit** |
| --- | --- | --- | --- |
| **GJB2** | 760 | 338 | 422 |
| **109G＞A** | 399 | / | 399 |
| **235delC** | 288 | 270 | 18 |
| **299delAT** | 59 | 52 | 7 |
| **512insAACG** | 3 | / | 3 |
| **35delG** | 3 | 3 | 0 |
| **176del16** | 13 | 13 | 0 |

**Note:** Among the newborns screened for deafness genes using the 23-variant Kit, a total of 5 cases of compound heterozygous mutations were identified: 4 cases of GJB2 235delC with GJB2 109 G>A and 1 case of GJB2 299delAT with GJB2 109 G>A.

**Table S3** **Gene Screening Results of 25 Newborns Who Failed the Hearing Screening**

|  | **Hearing Screening**  **(unilateral or bilateral )** | **Genetic Screening(Kit)** | **WES result** |
| --- | --- | --- | --- |
| **Case 1** | Unpassed (bilateral) | Negative (9-variant Kit) | STRC c.2303_2313+1del12 heterozygote with Exon 16-19 duplication |
| **Case 2** | Unpassed (bilateral) | Negative (9-variant Kit) | GJB2 109G＞A homozygote |
| **Case 3** | Unpassed (bilateral) | GJB2 235delC heterozygote (9-variant Kit) | GJB2 c.235delC with GJB2 c.508_511dupAACG |
| **Case 4** | Unpassed (bilateral) | Negative (9-variant Kit) | KAT6B c.5385C>A heterozygote |
| **Case 5** | Unpassed (bilateral) | Negative (9-variant Kit) | Negative |
| **Case 6** | Unpassed (unilateral) | Negative (9-variant Kit) | / |
| **Case 7** | Unpassed (bilateral) | Negative (9-variant Kit) | / |
| **Case 8** | Unpassed (unilateral) | Negative (9-variant Kit) | / |
| **Case 9** | Unpassed (bilateral) | Negative (9-variant Kit) | / |
| **Case 10** | Unpassed (bilateral) | Negative (9-variant Kit) | / |
| **Case 11** | Unpassed (bilateral) | Negative (9-variant Kit) | / |
| **Case 12** | Unpassed (bilateral) | Negative (9-variant Kit) | / |
| **Case 13** | Unpassed (bilateral) | GJB2 235delC homozygote (9-variant Kit) | / |
| **Case 14** | Unpassed (bilateral) | Negative (9-variant Kit) | / |
| **Case 15** | Unpassed (bilateral) | Negative (9-variant Kit) | / |
| **Case 16** | Unpassed (unilateral) | Negative (9-variant Kit) | / |
| **Case 16** | Unpassed (bilateral) | Negative (9-variant Kit) | / |
| **Case 17** | Unpassed (unilateral) | Negative (9-variant Kit) | / |
| **Case 18** | Unpassed (bilateral) | GJB2 109G＞A homozygote (23-variant Kit) | / |
| **Case 19** | Unpassed (bilateral) | GJB2 109G＞A homozygote (23-variant Kit) | / |
| **Case 20** | Unpassed (unilateral) | GJB2 109G＞A homozygote (23-variant Kit) | / |
| **Case 21** | Unpassed (bilateral) | GJB2 c.235delC with GJB2 109G＞A  (23-variant Kit) | / |
| **Case 22** | Unpassed (bilateral) | GJB2 109G＞A homozygote (23-variant Kit) | / |
| **Case 23** | Unpassed (bilateral) | GJB2 109G＞A homozygote (23-variant Kit) | / |
| **Case 24** | Unpassed (bilateral) | GJB2 109G＞A homozygote (23-variant Kit) | / |
| **Case 25** | Unpassed (bilateral) | GJB2 109G＞A homozygote (23-variant Kit) | / |

“/” indicates that whole‑exome sequencing (WES) was not performed.
